# Supplementary material for: Isolation and characterization of plant-derived exosome-like nanoparticles from Carica papaya L. fruit and their potential as anti-inflammatory agent
Source: PLoS One. 2024 Jul 3;19(7):e0304335. doi: 10.1371/journal.pone.0304335 (PMC11221653; doi:10.1371/journal.pone.0304335)
Supplement: S1 Table — (PDF) [file pone.0304335.s001.pdf]

S1 Table. The Z-average, zeta potential, and PDI of the papaya PDEN stored at 4°C and −20°C for 0, 1, 2, and 4 weeks were analyzed by DLS

		Week				
Temperature	Indicator	0	1	2	3	4
4 ℃	Z-Average (nm)	168.8 ± 9.62	284 ± 3.3	271.6 ± 10.8	253.2 ± 5.3	241.6 ± 1.2
	PDI	0.231	0.207	0.261	0.270	0.404
-20 ℃	Z-Average (nm)	168.8 ± 9.62	200.1 ± 0.6	194.8 ± 3.3	190.4 ± 7.5	210 ± 2.9
	PDI	0.231	0.288	0.330	0.300	0.261
