# Supplementary material for: Isolation and characterization of plant-derived exosome-like nanoparticles from Carica papaya L. fruit and their potential as anti-inflammatory agent
Source: PLoS One. 2024 Jul 3;19(7):e0304335. doi: 10.1371/journal.pone.0304335 (PMC11221653; doi:10.1371/journal.pone.0304335)
Supplement: S1 Fig — (A) Freshly isolated, (B) 1 week, (C) 2 weeks, (D) 3 weeks, and (E) 4 weeks. Chromatograms show the most abundant GC-MS peaks. (PDF) [file pone.0304335.s002.pdf]

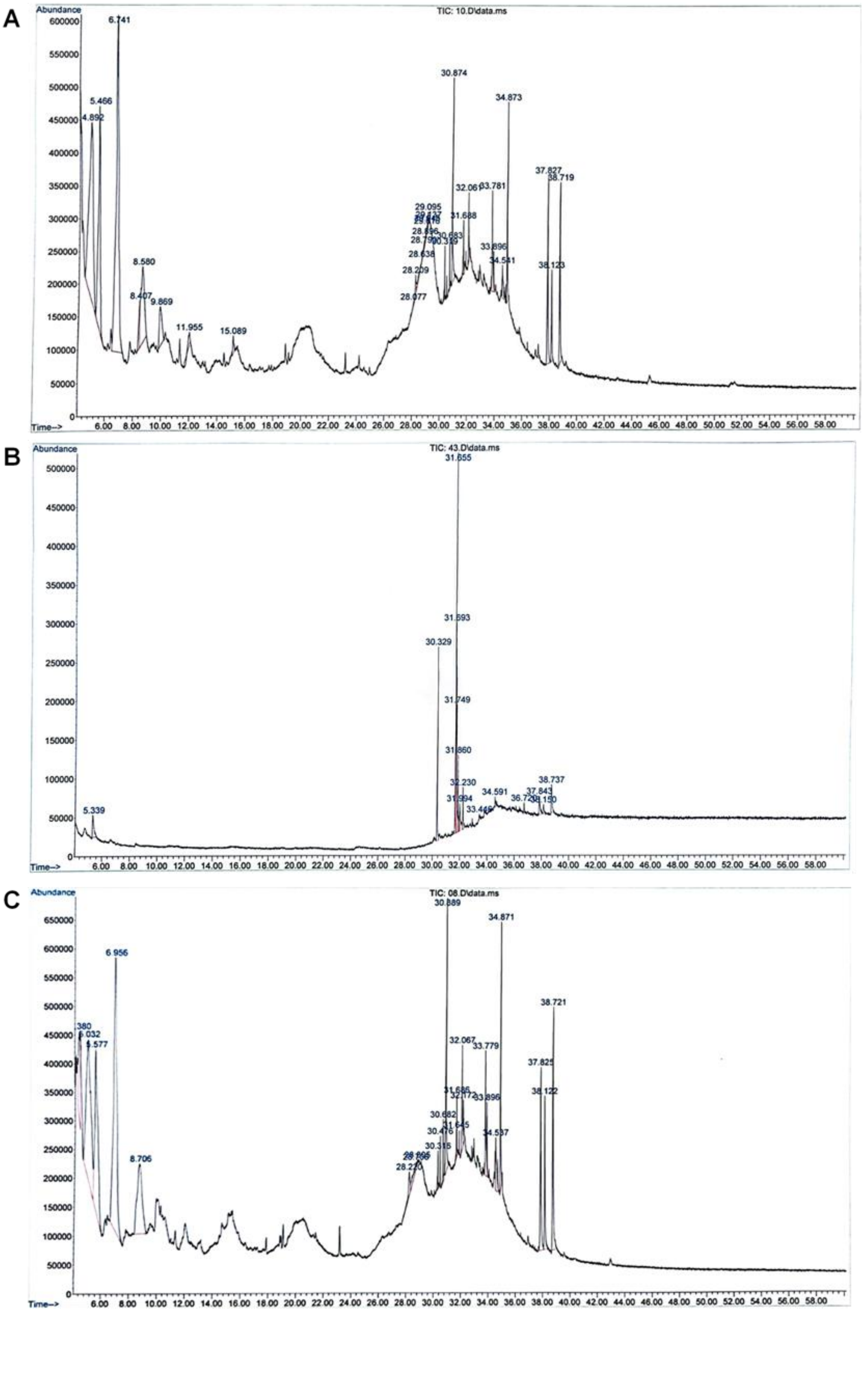
A
Abundance
TIC: 10.D\data.ms
6.741
5.466
4.892
8.580
8.407
9.869
11.955
15.089
30.874
34.873
37.827
38.719
32.061
33.781
29.095
31.688
28.896
28.638
28.209
28.077
33.896
34.541
38.123
Time-->
B
Abundance
TIC: 43.D\data.ms
31.655
31.593
30.329
31.749
31.860
32.230
38.737
34.591
37.843
5.339
C
Abundance
TIC: 08.D\data.ms
30.889
34.871
6.956
38.721
8.706
32.067
33.779
37.825
38.122
33.896
30.682
30.476
30.316
34.537
28.220
5.577
Time-->

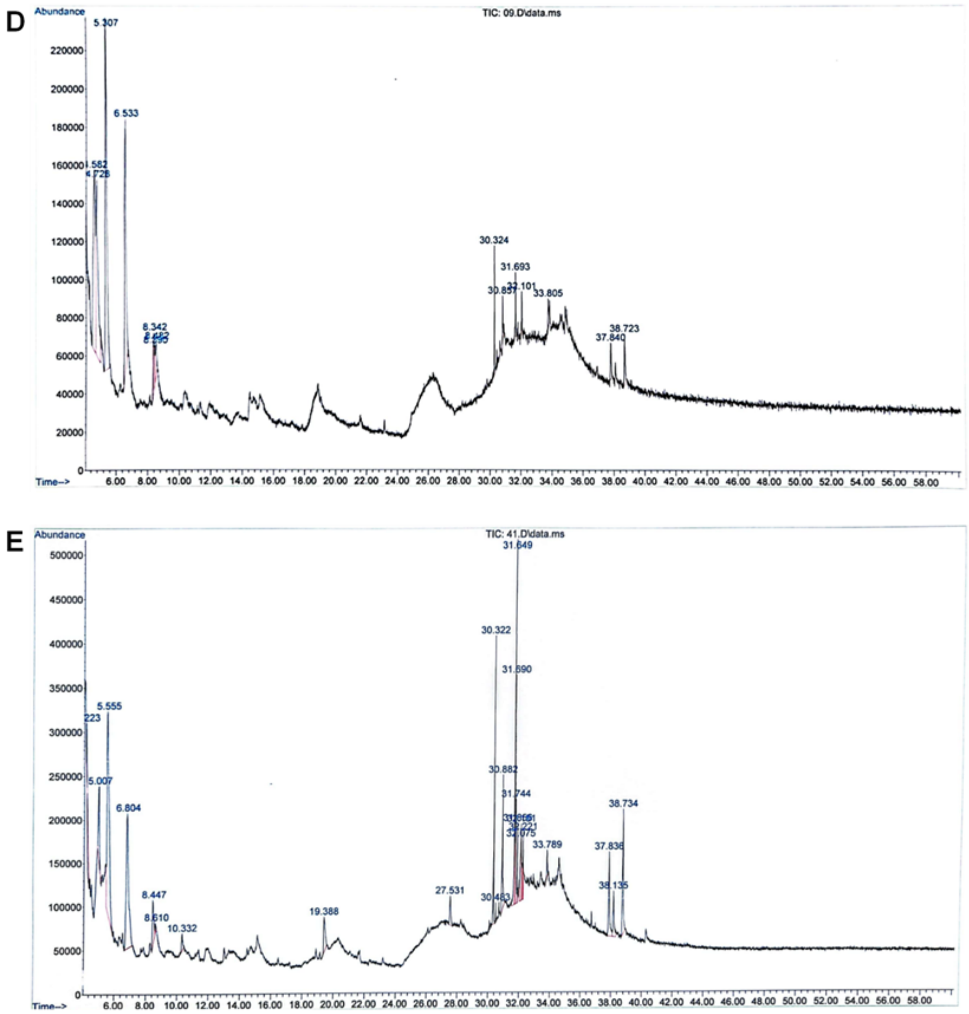

S1 Fig. Metabolite profiles of Papaya PDEN with different periods of storage

(A) Freshly isolated, (B) 1 week, (C) 2 weeks, (D) 3 weeks, and (E) 4 weeks. Chromatograms show the most abundant GC-MS peaks.
