# Supplementary material for: Isolation and characterization of plant-derived exosome-like nanoparticles from Carica papaya L. fruit and their potential as anti-inflammatory agent
Source: PLoS One. 2024 Jul 3;19(7):e0304335. doi: 10.1371/journal.pone.0304335 (PMC11221653; doi:10.1371/journal.pone.0304335)
Supplement: Attachment — Submitted filename: renamed_9b1ac.docx [file pone.0304335.s003.docx]

**Author’s Responds to Reviewers**

Title: Isolation and Characterization of Plant-Derived Exosome-Like Nanoparticles from *Carica papaya* L. Fruit and Their Potential as Anti-Inflammatory Agent

| **Reviewer’s Evaluation** | **Details of Author’s Respond** | **Modification** |
| --- | --- | --- |
| **Reviewer 1 Comments** | **Author’s Respond** |  |
| Kindly cite examples of natural products with anti-inflammatory potential, specifically downregulating the NF-Kb pathway (DOI: 10.1021/acsomega.2c06451) and PDE4 B2B enzyme (DOI: 10.1021/acsomega.1c00137) | Thank you for the suggestion. | We have added the citation in line 635-640. |
| The quality of the figures is poor; kindly upload high-quality ones | Thank you for the suggestion. | We have changed the figure with 300 DPI resolution |
| Table 4 identified the top-ranked metabolites present in the tested PDEN preparations. Given the current state of the paper, I believe it is not yet suitable for publication in PLOS One as it lacks rigor and in-depth analysis, especially in the biological assay part. To bridge this gap, I would require the authors to perform in silico analysis via molecular docking and computational pharmacokinetic evaluation, utilizing SWISS ADME and OSIRIS Toxicity software to further characterize the biological potential of the top-ranked compounds. Since the study hypothesized that the anti-inflammatory effects have something to do with the inhibition of the NF-Kb pathway, it is prudent to target the different enzymes and proteins from this pathway. For the computational analyses, please follow the protocols from these papers: DOI: 10.3390/computation10010007, DOI: 10.1080/07391102.2021.1969281 | Thank you for the suggestion.  To identify the compounds responsible for plant-derived exosome-like nanoparticle (PDEN) bioactivity, here we analyzed the metabolites profiles of PDEN isolated from papaya fruit with different ages; 0, 1, 2, 3, and 4 weeks. GC-MS-based metabolomics study reveals distinct profiles for these ages of papaya PDEN. Our study represent a step forward in the analysis of metabolites content of papaya fruit PDEN and it shows that papaya PDEN can be rich source of lead molecules in drug discovery based on their capability to store and transport unique and diverse chemical structures. Our findings reveal that papaya PDEN transport metabolite cargo with therapeutic interest. Taken together, the present study was the first to isolate and characterize PDENs from papaya fruit. Our study employs a GC-MS method consistent with that utilized in previous research DOI: 10.1371/journal.pone.0296259  Therefore, affirming the suitability of our data for publication in this journal.  This study aimed to show the potential of papaya PDEN cargoes in the therapeutic realm, such as its potential as an anti-inflammatory agent. However, the potential of each cargo contained in papaya PDEN was only enriched through literature studies that utilized related compounds, which is the limitation of this study. In consequence, further studies in the next research are needed to explore the mechanisms of anti-inflammatory effects associated with metabolites found in this GC-MS analysis. Study with LC-MS/MS will be prepared for our future study in elucidating the content of PDEN especially related to anti-inflammatory properties. |  |
| At this point, there is no sufficient evidence to support the proposed mechanisms shown in Figure 12 even though they specifically mentioned it's only hypothetical, as no further assays were performed to specifically target NF-Kb and IL-10-related JAK-STAT machineries. I suggest excluding this figure | Thank you for the suggestion. | We have excluded hypothetical mechanism figure. |
| Figure 11 should include tissue photomicrographs of the zebrafish used as the animal model | Thank you for the suggestion. | We have added images of zebrafish as animal model. |
| The study is limited only to qRT-PCR data, which only shows mRNA expression. However, mRNA expression does not always correlate with protein expression. Please justify why western blot analysis was not carried out to investigate protein expression | Thank you for the suggestion.  We have carried out an analysis with an in vivo animal model of zebrafish to see the ability of papaya PDEN as an anti-inflammatory agent. The results have shown that papaya PDEN is able to inhibit the migration of neutrophils and macrophages to the wound site. We assume that the interleukin protein that we analyzed was expressed because there was a decrease in migration in zebrafish treated with varying PDEN papaya concentrations, whereas in the negative control (without any treatment) there was higher migration. Therefore we did not perform western blot because both qRT-PCR and zebrafish model have shown papaya PDEN ability as anti-inflammation agent |  |
| For the RT-PCR assay, kindly include the image of the gel to further showcase the effects of PDENs on the expression of target genes | Thank you for the suggestion.  We think the image of the gel is not required because the qRT-PCR method is a highly sensitive technique which is capable of quantifying the expression of the target gene present in a sample with high precision. |  |
| What are the limitations of the present study? Please provide recommendations to address these limitations | The limitation of this study are:   1. This study represents an analysis of the metabolite content of papaya PDEN and shows that papaya PDEN have potential as anti-inflammation agent. However, the potential utilization of metabolites found in papaya PDEN in this study have only been carried out through literature studies, so further studies are needed to prove the ability of papaya PDEN as anti-inflammation agent. 2. This study focuses on the ability of papaya PDEN to be internalized in RAW 264.7 cell cultures. However, this study does not specifically prove the internalization mechanism of PDEN in papaya fruit, so further studies are needed to determine the uptake mechanism in target cell cultures. 3. This study focuses on the ability of papaya PDEN as anti-inflammation agent only on mRNA level and using amputated caudal fin of zebrafish larvae model. Further studies are needed to determine the anti-inflammation ability of papaya PDEN. |  |
| **Reviewer 2 Comments** |  |  |
| In abstract, Please provide the full name of DLS, TEM, BCA | Thank you for the suggestion. | We have provided the full name of DLS, TEM, BCA. |
| Please replace keywords that are in the title, basic rule | Thank you for the suggestion. | We have replaced the keywords. |
| The manuscript needs another revision to check for spacing (spaces after words and before the citations are often missing) | Thank you for the suggestion. |  |
| **Reviewer 3 Comments** |  |  |
| Kindly state the post-hoc test in the statistical analysis | Thank you for the suggestion. | We have stated the post-hoc test in the statistical analysis. |
| It is much appreciated if the images provided with the scale bar and measurement within the image | Thank you for the suggestion. | We have provided scale bar and measurement at the bottom right of the images. |
| Figure 1 and 2 somehow are blurred | Thank you for the suggestion. | We have changed the figure with 300 DPI resolution |
| It is strongly recommend the images of zebrafish for the neutrophils and macrophages counts | Thank you for the suggestion. | We have added images of zebrafish for the neutrophils and macrophage |
| Kindly provide reference for line 78-79 | Thank you for the suggestion. | We have provided the references. |
| Typo at line 570, degree celcius double "C" | Thank you for the suggestion. | We have fixed the typo. |
